# In-store marketing of inexpensive foods with good nutritional quality in disadvantaged neighborhoods: increased awareness, understanding, and purchasing

**DOI:** 10.1186/s12966-016-0427-1

**Published:** 2016-09-27

**Authors:** Axel Gamburzew, Nicolas Darcel, Rozenn Gazan, Christophe Dubois, Matthieu Maillot, Daniel Tomé, Sandrine Raffin, Nicole Darmon

**Affiliations:** 1 UMR “Nutrition Physiology and Ingestive Behavior”, AgroParisTech, INRA, Université Paris-Saclay, F-75005 Paris, France; 2 MS-Nutrition, Aix-Marseille Université, Marseille, France; 3 UMR “Nutrition, Obesity and Risk of Thrombosis”, INRA, INSERM, Aix-Marseille Université, 13385 Marseille, France; 4 LinkUp, Levallois-Perret, France

**Keywords:** Intervention, Low-income, Nutrient profiling, Price, Social marketing, Exploratory study, Nutrition information, Shelf labeling, Sales

## Abstract

**Background:**

Consumers often do not understand nutrition labels or do not perceive their usefulness. In addition, price can be a barrier to healthy food choices, especially for socio-economically disadvantaged individuals.

**Method:**

A 6-month intervention combined shelf labeling and marketing strategies (signage, prime placement, taste testing) to draw attention to inexpensive foods with good nutritional quality in two stores located in a disadvantaged neighborhood in Marseille (France). The inexpensive foods with good nutritional quality were identified based on their nutrient profile and their price. Their contribution to customers’ spending on food was assessed in the two intervention stores and in two control stores during the intervention, as well as in the year preceding the intervention (*n* = 6625). Exit survey (*n* = 259) and in-depth survey (*n* = 116) were used to assess customers’ awareness of and perceived usefulness of the program, knowledge of nutrition, understanding of the labeling system, as well as placement-, taste- and preparation-related attractiveness of promoted products. Matched purchasing data were used to assess the contribution of promoted products to total food spending for each customer who participated in the in-depth survey.

**Results:**

The contribution of inexpensive foods with good nutritional quality to customers’ total food spending increased between 2013 and 2014 for both the control stores and the intervention stores. This increase was significantly higher in the intervention stores than in the control stores for fruits and vegetables (*p* = 0.001) and for starches (*p* = 0.011). The exit survey revealed that 31 % of customers had seen the intervention materials; this percentage increased significantly at the end of the intervention (*p* < 0.001). The in-depth survey showed that customers who had seen the intervention materials scored significantly higher on quizzes assessing nutrition knowledge (*p* < 0.001) and understanding of the labeling system (*p* = 0.024).

**Conclusion:**

A social marketing intervention aimed at increasing the visibility and attractiveness of inexpensive foods with good nutritional quality may improve food purchasing behaviors in disadvantaged neighborhoods.

## Background

European Food Information for Consumers regulations [1] mandate nutrition declarations (energy value, amounts of fat, saturates, carbohydrate, sugars, protein and salt) for most pre-packed foods and allows for the voluntary inclusion of simplified nutrition labels to make information more visible and understandable at the time of purchase. Several studies have attempted to identify the nutrition labels that are the most informative and useful to consumers. These studies mostly focused on the perception and/or the understanding of these labels, and were based on declarative methods (questionnaires) [2–7]. Few studies assessed the impact of nutrition labels on food choices, and these studies were either based on declarative methods (questionnaires) or experimental conditions (simulation of choices on computer/printouts) [8–10]. It is thus necessary to assess customers’ perception and understanding of nutrition labels in real-life conditions and determine the resulting impact on actual food choices [11].

Several in-store interventions promoting healthy foods using a simplified nutrition labeling system have now been conducted in different countries [12–30]. Such research suggests that many consumers pay attention to nutrition labels and these labels can affect knowledge, attitudes and intentions [12, 14, 15, 19–22, 25, 26], however, the effect on purchasing behaviors may be more limited. Although some recent studies have demonstrated encouraging results on customers purchases or store sales [23, 24, 27–30], reviews of the literature have found more mixed results and the overall effect of nutrition labels on purchasing behaviors is inconsistent over studies [31–33].

Several reasons may explain why nutrition information, even when it is simplified, not always has the expected impact. First, although many consumers have a positive attitude towards nutrition information, it has been argued that some consumers perceive nutrition labels as too complicated and have a limited understanding of the information [2, 3]. Understanding may also differ greatly between different consumers. A study conducted in six European countries suggests that understanding of nutrition information differs significantly between countries [22], with high understanding found in the UK, Sweden and Germany, and more limited understanding found in France, Poland and Hungary. Second, food purchases are usually influenced by many factors other than nutrition concerns [34, 35]. Even when nutrition information is well understood by the customers, the foods that are recommended nutritionally can be perceived as unpalatable or unsatisfying, may not be compatible with cultural or family standards, and may be more expensive and require increased knowledge, skills, time and effort to prepare [36, 37]. Accordingly, interventions were more likely to be effective when they combined nutrition labeling with additional intervention components to increase the visibility and attractiveness of healthy foods in stores [31–33]. These additional intervention components may be mass-media campaigns outside the store [15–17], in-store advertising [12, 15, 17–21, 24, 27, 28], placement strategies [25, 27, 28], taste testing [14, 18–21, 28] and/or cooking demonstrations [14, 18, 19, 21]. Third, the decision-making processes of making purchases are strongly affected by in-store environmental cues and time pressure [38–40]. The average magnitude of effect attributable to an intervention is often modest in comparison with other factors influencing consumer purchases such as price variations in the stores, season, or socio-economic status of target population [13], highlighting the need for powerful evaluation designs to assess the effectiveness of programs that operate in the context of many other influences.

In order to develop multicomponent interventions with understandable nutrition information in a real-world setting, the use of marketing principles and techniques has been increasingly advocated in the past few decades [41–45], with the idea of using so-called “social marketing” to help positively influence behavior. Although social marketing campaigns are developed based on evidence from the health and behavioral literature, a key feature of these interventions is creating clear, useful, and salient messages by conducting preliminary exploratory research and pre-testing material [45, 46]. This type of preliminary research often uses qualitative data such as focus groups and in-depth interviews to explore the values, perceptions, aspirations, and concerns of the target population to understand what factors reach individuals and ultimately motivate them to change their behavior.

The lower the socio-economic status of an individual, the less likely he or she is to understand and implement untargeted public health information and policies, which is suspected to contribute to the aggravation of social inequalities in health [47]. In particular, nutrition declarations and nutrition labels are perceived as too complex, and ethnic minority populations and populations with a low socio-economic status are less likely to understand them [48, 49]. It is therefore necessary to undertake campaigns that specifically target populations with a low socio-economic status, adapting the message to these populations and taking into account the skills it requires to them for interpretation.

It is difficult to maintain a balanced diet on a small budget, and can be impossible if the amount of money dedicated to food per day and per person falls below a certain level [50]. However, for individuals whose spending capacity falls close to, but above, this threshold, it is possible to maintain a balanced diet by selecting foods with good nutritional quality for their price [51]. Based on this premise, the OPTICOURSES project [52] was implemented in disadvantaged neighborhoods in Marseille (France) to attempt to improve the nutritional quality of food purchases in populations with budgetary constraints. The project addressed both the demand (participatory workshops) and the supply (in-store intervention), as well as the advantages of, and the interest in, inexpensive foods with good nutritional quality [53]. The objective of the present study was to evaluate the OPTICOURSES in-store intervention, which combined shelf labeling with a social marketing strategy to promote inexpensive foods with good nutritional quality.

## Methods

### Preliminary study

The 14th and 15th districts of Marseille are disadvantaged neighborhoods, in which 29.2 % and 28.1 % of people, respectively, were unemployed (France: 17.0 %) and 37.2 % and 41.3 % of people, respectively, did not have any higher education (France: 21.8 %) in 2012 [54]. To understand the factors that motivate food purchases in this population, we relied on results from the previous phase of the OPTICOURSES project. This previous phase was conducted in social centers (public locations where inhabitants of the neighborhood tend to gather for group activities, social support, public information, and other purposes) located in the 14th and 15th districts of Marseille from 2012 to 2014, and involved a series of participatory workshops on healthy eating and shopping for healthy food on a budget [52]. In order to gain a better understanding of the factors that promote and inhibit healthy eating in our target population, we conducted in-depth interviews with 16 of the workshop participants (data not shown). Consistent with the existing literature [55], the main conclusions from this preliminary study were as follows:

- □ Price is a major concern for this population when making food choices, and is in fact one of the most important determinants of food purchases.
- □ Taste is also an important food choice determinant in this population, especially when it comes to pleasing children.
- □ This population has an intuitive understanding of the concept of a quality/price ratio, although the term “quality” was used to describe many different aspects.
- □ There is a strong association between concerns about diet quality and cooking practices and meal preparation in this population.

In addition to these workshops and interviews, exploratory work combining geographical and sociological analyses in the 14th and 15th districts of Marseille showed that residents of these neighborhoods preferentially shop at local discount stores for their food purchases [56].

### Setting

Discount stores located in the 14th and 15th districts of Marseille and belonging to the same retail chain (DIA) were contacted for the intervention. These stores sell food products at low prices, and are often relied on by local residents as a primary, local source for food purchases. Four stores were identified by the retail chain managers as being comparable in terms of size, number of employees, type of supply, whole store sales, and number of visits per day, and were therefore selected for the intervention. Two of the stores received a 6-month intervention (from January to June 2014) to promote inexpensive foods with good nutritional quality, and the two other stores served as assessment-only controls. The stores were assigned to intervention/control arms based on schedule considerations of the retailer. Approximately 1.5 miles separated intervention stores from each other, and the two intervention stores were separated from the two control stores of at least 5 miles, in another district, making it very unlikely that customers shop at both tests and control stores. All four stores belonged to the same retail chain (DIA), had 5 to 10 employees, and had less than 1,000 square feet of floor space. They also had similar planograms (shelf organization), product offerings (about 3,000 items), and prices. They were open from Monday to Saturday from 9 am to 7 pm and experienced between 200 and 300 visits per day. These stores had also a loyalty card system recording member-customers’ purchases.

### Promoted products

Each food product on offer in the stores was matched to a corresponding generic food in the French food composition database associated with the INCA 2 national food consumption survey [57]. We then identified the foods to be promoted during the in-store marketing intervention based on three criteria. Hence, the promoted products had to:

1. Be of “good nutritional quality”, defined as having a SAIN/LIM ratio [51, 58] above the median for the 1,304 foods included in the INCA 2 French food composition survey;
2. Be “inexpensive”, defined as having a selling price below the first tertile of the mean national price of foods from the same food category in the French database, as previously described [59];
3. Be a “source of” at least two nutrients, according to European regulations on nutritional claims [60].

The SAIN,LIM system was developed by Darmon et al. [58] based on their content in protein, fiber, calcium, vitamin C, iron, saturated fatty acids, added sugars, and sodium. The SAIN/LIM ratio has previously been shown to correlate well with modeled diets that meet a full set of nutrient recommendations [51]. Product information was obtained from the product packages or in collaboration with the retail chain quality managers. All products meeting the three criteria were promoted. As prices were variable, the products promoted in the two intervention stores were not strictly identical, and also varied slightly during the 6-month intervention. On average, 180 products from almost all food categories were selected, including items such as milk, plain yoghurt, eggs, canned or frozen fruits and vegetables, canned or frozen fish, soups, pulses, fruit compotes and juices, plus approximately 90 fresh fruits and vegetables.

### Intervention

**Fig. 1:**
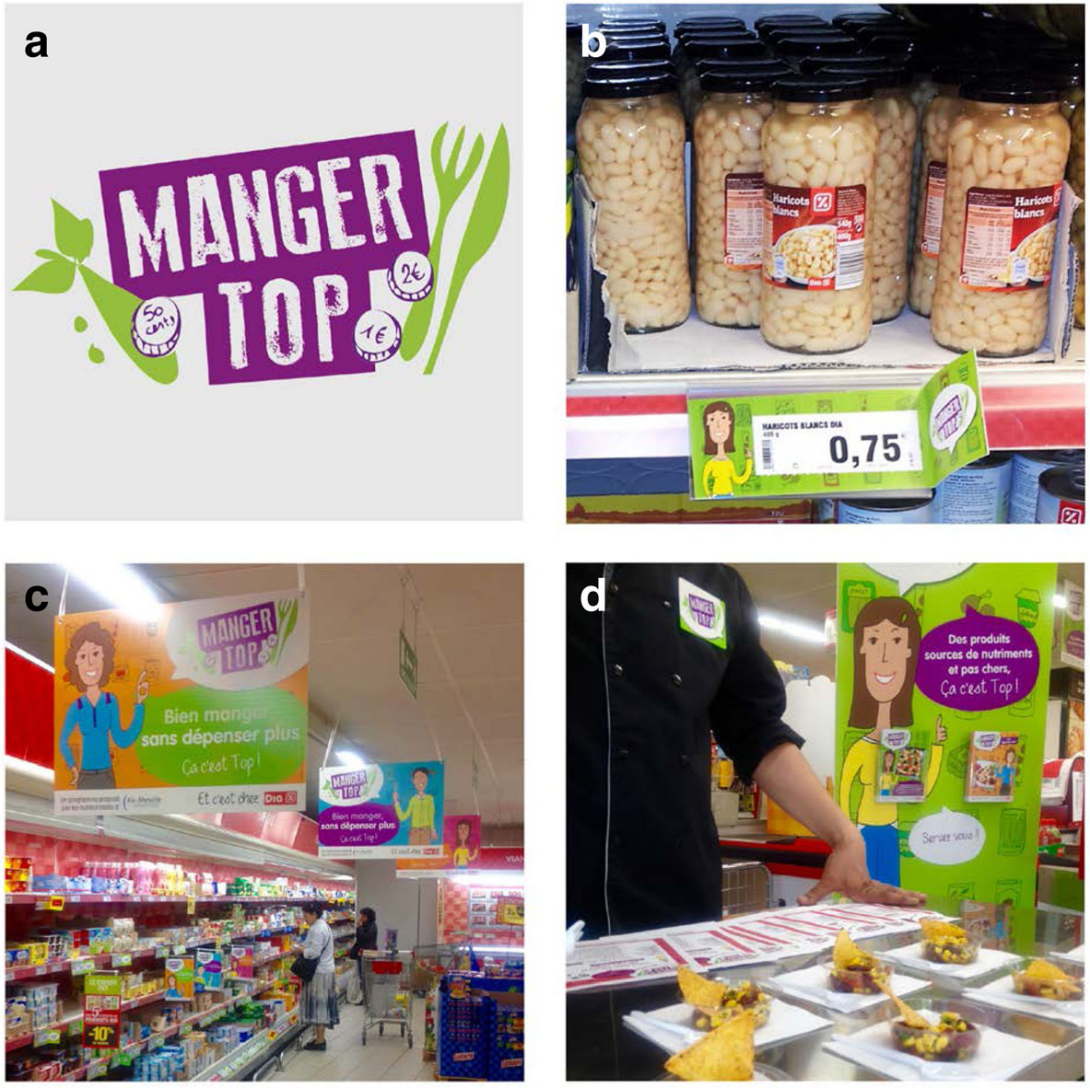
Elements of the intervention. **a** MANGER TOP logo, **b** Shelf labeling system for promoted foods, **c** In-store posters of MANGER TOP intervention, **d** In-store taste-testing booth of MANGER TOP recipes

The intervention was designed to meet the concerns of the target population, which were identified during the preliminary exploratory study. The name of the intervention-MANGER TOP (“eating great”)-was designed to carry a positive message about food, while remaining simple and non-institutional. The MANGER TOP intervention consisted of three complementary promotion strategies: 1) shelf labels for all of the products in the store that had been identified as inexpensive foods with good nutritional quality; 2) posters and leaflets with the MANGER TOP logo explaining the labeling system; and 3) prime placement, a taste-testing booth, and leaflets specifically focused on canned fish, pulses, and eggs, as they are three food families that are known to be inexpensive and have good nutritional quality [51]. The MANGER TOP materials have been developed in close collaboration with an advertising agency. The creative department defined messages and communication materials that meet the objectives of the intervention, seeking them to be adapted to the context and the target populations. As consumers often do not use nutrition information in a real-world setting, the intervention tools were based on positive aspects such as appetizing pictures of foods, preparation tips and recipes, together with simplified nutritional information (see Fig. 1). The overall objective was to increase the visibility and attractiveness of inexpensive foods with good nutritional quality. There were no reductions in the price of the promoted products. All of the intervention materials were improved after a pre-test that was conducted with a group of residents from the neighborhood. The intervention materials were introduced on a gradually increasing basis to gain momentum in the awareness of customers. The visual identity of MANGER TOP campaign was introduced in January 2014 while the labeling system started in February, and the taste-testing booth was initiated in April. This was made to revive customers’ awareness and interest for the MANGER TOP program and to avoid boredom feeling towards the intervention materials after the first months.

The intervention strategies were developed in consultation with the store operators and retail chain managers in order to ensure consistency with the stores’ constraints and opportunities. However, the store staff was not involved in the implementation of the intervention, which was totally performed by the research team, which was regularly in the stores to install, follow up, and adapt the intervention materials, and could therefore answer customer questions. The inte﻿rvention was evaluated through purchasing data analysis, as well as exit surveys and in-depth surveys admistrated to the store customers (see Table 1).

**Table 1:** Summary of the evaluation protocol

Method	Duration	Place	Period	Sample	Customers
Purchasing data	-	2 intervention stores and 2 control stores	January to June 2013 (baseline) and January to June 2014 (intervention)	6625 customers	Member-customers only, making 100 % of their purchases in the same store, with at least one purchase during baseline and during intervention
Exit surveys	2 min	2 intervention stores	May-June 2014	259 customers	All customers
In-depth surveys	15 min	2 intervention stores	June 2014	116 customers	Member-customers only

### Purchasing data

Detailed monthly purchasing data for member-customers (*n* = 11,281) were collected from the two intervention stores and the two control stores during the intervention period (January to June 2014) and in the year preceding the intervention (January to June 2013). Data from 2013 and 2014 were collected during the same months, avoiding any potential seasonal effect. Only customers who had made 100 % of their loyalty card recorded purchases in the same store (among the four stores) and who purchased at least one food product during each of the two periods (2013 and 2014) were selected, resulting in a sample size of 6,625 customers.

### Exit surveys and in-depth surveys

During the final two months of the intervention period, we conducted exit surveys and in-depth surveys with customers leaving the two intervention stores. The exit surveys and the in-depth surveys were administered at the intervention stores in two independent groups of customers. To obtain a cross-section of all potential shopper profiles, the surveys were conducted on several different days and at several different times. The in-depth survey questionnaires were co-built and validated with a committee of experts in sociology and public health. The questions were inspired by the psychosocial variables measured by Gittelsohn and al. [26, 61] and the conceptual model proposed by Grunert and al. for studying effects of nutrition labels on consumers [3]. These questions were translated into French and adjusted to the objectives of our study. The in-depth survey questionnaires went through several versions, were pre-tested with 21 customers and adapted for a low literacy population.

All customers leaving the store were approached and asked to complete an exit survey, which took approximately 2 min to complete. For the in-depth survey all customers leaving the store were approached, but only member-customers aged 18 years or more and speaking French could participate. On one day of survey, approximately 80 to 100 customers were approached. Of these, about 50 % did not meet the criteria (no loyalty card or under 18), about 25 % refused to participate (lack of time or other reasons), and about 25 % participated to the survey; each survey lasted approximately 15 min, and the member-customers received a €10 voucher (that they can use in the store or in others stores) as an incentive for participation.

### Primary outcome measures

#### Purchasing data

The food products were classified according to six food categories: fruits and vegetables (such as fresh or processed vegetables, fresh or processed fruits, fruits juices, nuts, and soups), starches (such as breakfast cereals, legumes, breads, potatoes, pasta, and rice), meat/fish/eggs (including deli products), mixed dishes and sandwiches, dairy products (such as cheese, milk, and fresh dairy products) and other. The “other” food category included foods for which very few products were promoted, mainly due to having a SAIN/LIM ratio lower than the median; these included fatty/salty/sugary products, fat products, seasonings, water, and other beverages. Fresh fruits and vegetables that benefited from one-time, limited discounts were not promoted. For each member-customer in control stores and in intervention stores, we calculated the contribution of promoted products to the total spending on food and to the spending by food category for the intervention period (2014) and the baseline period (2013). If the customer did not purchase any product within a food category during a particular year, we treated this as a missing value. Purchasing data from January were excluded because the labeling system was not yet fully in place.

#### Exit surveys

A total of 259 individual exit surveys were conducted over three periods of time (April 29 to May 15, May 19 to June 12, and June 13 to 24) in the two intervention stores. During the survey, customers were asked if they had seen the intervention materials as they were shopping at the store (awareness of the program) and if they considered such a program to be useful (perceived usefulness of the program).

#### In-depth surveys

A total of 144 in-depth surveys were conducted from May 20 to June 26 in the two intervention stores. We used loyalty card numbers, which were collected during the survey, to match the customers’ survey responses to their purchasing data. Twenty-eight customers could not be matched with their purchasing data and were excluded from the sample, leaving a final sample size of 116 customers. During the survey, customers were asked if they had seen the intervention materials as they were shopping at the store (awareness of the program) and if they considered such a program to be useful (perceived usefulness of the program). They also took a quiz assessing their knowledge of nutrition and a quiz assessing their understanding of the labeling system. In addition, we showed each customer eight promoted products and asked if these products were easy to find in the store (placement-related attractiveness), if they considered these products to be appetizing (taste-related attractiveness), and if they would be able to prepare a meal with these products (preparation-related attractiveness). We summarized the answers to the quizzes and the questions on attractiveness using a scoring system, with a score on 100 for each of the following outcomes: knowledge of nutrition, understanding of the labeling system, placement-related attractiveness, taste-related attractiveness, and preparation-related attractiveness. For each customer, we used the matched purchasing data from their loyalty cards to calculate the contribution of promoted products to total food spending during the intervention, as well as shopping profile variables (frequency of shopping at the store, average spending in the store per month, average spending in the store per visit). We also collected socio-demographic information.

### Statistical analysis

#### Purchasing data

The average contribution of promoted products to the total spending on food, overall and by food category, were compared based on the type of store (intervention/control) and year (2013/2014) using a generalized linear mixed model. Each model included fixed effects for year (2013/2014), type of store (intervention/control), and store (four stores), which was treated as a nested effect within the type of store. The interactions between the type of store and year, as well as between the individual store and year, were also taken into account in the model. The interaction between the type of store and year allowed us to test whether the variation in the contribution of promoted products to overall purchases between 2013 and 2014 differed between the intervention and control stores, and therefore whether the intervention had a specific impact. A random effect for customer was included by using an unstructured covariance matrix, which accounted for the annual repeated measures.

#### Exit surveys

The *χ* ^2^ test was used to analyze differences in customers’ awareness and perceived usefulness of the program between the two intervention stores and between three different survey periods, all of which started when all the marketing materials were in place (April 29 to May 15, May 19 to June 12, and June 13 to 24).

#### In-depth surveys

Fisher tests were used to analyze differences in customers’ awareness and perceived usefulness of the program (categorical variables) based on socio-demographic characteristics and shopping profiles. Differences in customers’ scores and spending on promoted products (continuous variables) based on socio-demographic characteristics and shopping profiles were analyzed by one-way ANOVAs. One-way ANOVAs were also performed to determine whether the continuous variables (scores and spending on promoted products) were associated with customers’ awareness and perceived usefulness of the program. We also performed the Kruskal-Wallis nonparametric test on skewed data to confirm the ANOVA findings. Pearson correlation tests were conducted to determine whether spending on promoted products correlated with the scores.

Analysis of the purchasing data was performed using SAS version 9.4 (SAS Institute), and the other analyses were performed using R version 3.1. All tests were based on a 0.05 significance level.

## Results

### Purchasing data

As shown in Table 2, the average contribution of inexpensive foods with good nutritional quality to customers’ total spending on food reached around 20 % in both the control and intervention stores. This contribution increased between 2013 and 2014 in both the control and the intervention stores (by 0.6 % and 1.4 %, respectively). There was no significant difference in the overall increase depending on the type of the store (*p* = 0.235 for the year/type of store interaction). However, the increase in purchases of inexpensive foods with good nutritional quality among fruits and vegetables and among starches was significantly greater in the intervention stores compared to the control stores (*p* = 0.001 and *p* = 0.011, respectively, for the year/type of store interaction), which suggests that the intervention had a positive impact on purchases for these two food categories. In fact, for these two food categories, baseline differences in purchases were observed between control and intervention stores-with significantly higher contributions of inexpensive foods with good nutritional quality in the control stores-but the differences no longer persisted after the intervention, showing the positive impact of the intervention on the purchase of promoted products. No significant differences were observed in the increase in purchases from the other food categories based on the type of store.

**Table 2:** Impact of the intervention on the purchases of inexpensive foods with good nutritional quality

Food category	Control stores (*n* = 3974)		Intervention stores (*n* = 2651)		P year	P type of store	P-interaction year*type of store
	2013	2014	2013	2014			
	Mean (SD)	Mean (SD)	Mean (SD)	Mean (SD)			
All	21.4 (12.0)	22.0 (13.8)	20.0 (11.7)	21.4 (14.7)	<0.001	<0.001	0.235
Fruits and vegetables	50.7 (18.9)	52.4 (21.5)	48.3 (20.7)	52.5 (24.1)	<0.001	<0.001	0.001
Starches	28.7 (26.0)	29.8 (28.1)	25.7 (24.6)	29.8 (29.3)	<0.001	0.000	0.011
Meat/Fish/Eggs	15.0 (23.0)	14.7 (24.9)	22.4 (29.9)	22.2 (32.1)	0.006	<0.001	0.363
Mixed dishes and sandwiches	1.2 (6.6)	1.3 (6.2)	1.3 (6.8)	1.7 (8.7)	0.009	0.031	0.056
Dairy products	19.5 (21.2)	19.8 (23.1)	23.1 (23.5)	22.7 (26.6)	0.867	<0.001	0.363
Others	0.4 (2.3)	0.6 (4.2)	0.6 (3.1)	0.8 (4.4)	0.015	0.010	0.297

Average contribution (%) of inexpensive foods with good nutritional quality to the total spending on food of member-customers, overall and by food category. Purchases of promoted products were compared based on the type of store (intervention/control) and year (2013/2014) using a generalized linear mixed model

### Exit surveys

#### Customers’ awareness of the program (data not shown)

In the exit surveys, 15 % of customers spontaneously mentioned that they had seen the intervention materials (spontaneous awareness), and an additional 16 % of customers recognized the materials after they were shown one of the intervention posters (aided awareness), resulting in a total awareness of 31 %. This percentage was significantly higher during the two last survey periods compared to the first survey period (*p* < 0.001): 24 % of total awareness for the first period (April 29 to May 15), and 35 % of total awareness for the two last periods (May 19 to June 12 and June 13 to June 24). No significant difference was observed between the two intervention stores.

#### Customers’ perceived usefulness of the program (data not shown)

After a brief explanation of the labeling program, 60 % of customers in the exit surveys replied that such a program could be useful when making purchases. This percentage was significantly higher in the last survey period compared to the first two survey periods (*p* < 0.001): 51 % of perceived usefulness for the first period (April 29 to May 15), 56 % of perceived usefulness for the second period (May 19 to June 12), and 74 % of perceived usefulness for the third period (June 13 to June 24). No significant difference was observed between the two intervention stores.

### In-depth surveys

#### Customers’ awareness of the program (data not shown)

In the in-depth surveys, 19 % of customers spontaneously mentioned that they had seen the intervention materials (spontaneous awareness), and an additional 25 % of customers recognized the materials after they were shown an intervention poster (aided awareness), resulting in a total awareness of 44 %. This percentage was significantly higher when the customers shopped at the intervention store more frequently (*p* = 0.037): 32 % of total awareness for customers shopping less than once a month in the store, 36 % of total awareness for customers shopping two to four time a month in the store, and 68 % of total awareness for customers shopping more than four times a month in the store. No significant differences were observed across other socio-demographic characteristics or shopping profiles.

#### Customers’ perceived usefulness of the program (data not shown)

After being provided with a brief explanation of the labeling program, 92 % of customers in the in-depth surveys replied that such a program could be useful when making purchases. No significant differences were observed across socio-demographic characteristics or shopping profiles.

#### Customers’ knowledge of nutrition

As shown in Table 3, customers who were aware of the intervention scored significantly higher for knowledge of nutrition, with a significant difference between spontaneous awareness and aided awareness (*p* < 0.001).

#### Customers’ understanding of the labeling system

As shown in Table 3, customers who were aware of the intervention and customers who considered the intervention to be useful scored significantly higher on understanding the labeling system (*p* = 0.024 and *p* = 0.007, respectively). Understanding the labeling system also correlated positively with knowledge of nutrition (*p* = 0.020, *r* = 0.22; data not shown).

#### Attractiveness of promoted products

As shown in Table 3, the score measuring the taste-related attractiveness of promoted products was significantly higher among older customers (*p* = 0.009) and customers in higher socio-professional categories (*p* = 0.028). The score measuring the preparation-related attractiveness of promoted products was significantly higher among women (*p* = 0.036) and customers who shopped two to four times a month at the store (*p* = 0.013).

**Table 3:** In-depth survey: knowledge in nutrition, understanding of the labeling system, and attractiveness of promoted products

	Sample	Knowledge of nutrition	P	Understanding of the labeling system	P	Placement-related attractiveness	P	Taste-related attractiveness	P	Cooking-related attractiveness	P
	(*n* = 116)	Mean (SD)		Mean (SD)		Mean (SD)		Mean (SD)		Mean (SD)	
Total	116	62.8 (20)		79.4 (15)		85.7 (22)		52.8 (13)		68.5 (20)	
Sex			0.310		0.319		0.143		0.258		0.036
Female	91	63.7 (20.6)		80.1 (14.9)		84.5 (23.5)		52.1 (12.7)		70.6 (19.0)	
Male	25	59.2 (15.8)		76.8 (13.5)		90.0 (14.0)		55.5 (15.7)		61.0 (23.5)	
Age			0.084		0.669		0.401		0.009		0.597
20–40 years	29	69.0 (18.2)		79.3 (13.6)		89.2 (20.5)		48.3 (6.9)		65.9 (20.6)	
40–60 years	51	58.8 (18.9)		78.2 (16.6)		86.3 (19.2)		51.3 (14.3)		70.6 (19.2)	
More than 60 years	36	63.3 (21.1)		81.1 (12.6)		81.9 (26.1)		58.5 (14.4)		67.7 (22.0)	
Adults at home			0.926		0.948		0.522		0.631		0.089
One	26	63.1 (20.2)		79.2 (15.2)		82.7 (28.3)		51.7 (13.8)		74.5 (23.0)	
Two or more	90	62.7 (19.7)		79.4 (14.6)		86.5 (19.8)		53.1 (13.3)		66.8 (19.3)	
Children at home			0.180		0.701		0.947		0.283		0.175
No child	45	64.9 (18.2)		80.7 (14.4)		85.8 (23.2)		55.0 (15.1)		64.2 (22.1)	
One or two	47	58.7 (20.6)		78.1 (16.6)		86.2 (20.2)		52.3 (11.4)		70.7 (18.8)	
Three or more	24	66.7 (20.1)		79.6 (10.8)		84.4 (23.4)		49.7 (13.6)		72.4 (19.1)	
Food insecurity			0.924		0.845		0.333		0.863		0.295
Yes	19	63.2 (15.3)		80.0 (10.0)		90.1 (18.0)		53.3 (14.0)		73.0 (22.5)	
No	97	62.7 (20.5)		79.3 (15.4)		84.8 (22.6)		52.7 (13.3)		67.7 (19.9)	
Occupational status			0.279		0.346		0.350		0.028		0.967
Unemployed, disabled, student	42	65.7 (19.9)		77.4 (14.7)		89.9 (19.0)		49.0 (10.3)		68.2 (20.3)	
Lower socio-professional category	30	57.3 (17.2)		78.0 (15.6)		81.7 (21.5)		51.5 (14.2)		68.3 (20.2)	
Upper socio-professional category	12	60.0 (19.1)		85.0 (16.8)		88.5 (15.5)		56.8 (16.1)		66.7 (21.5)	
Retired	32	65.0 (21.6)		81.3 (12.6)		82.8 (27.1)		57.6 (13.9)		69.9 (21.0)	
Frequency of shopping at the store			0.792		0.877		0.579		0.142		0.013
Less than once	19	65.3 (22.9)		80.5 (11.3)		89.5 (12.0)		55.6 (9.1)		67.1 (19.2)	
Two to four times	66	61.8 (21.0)		79.5 (15.0)		82.4 (26.4)		50.8 (12.7)		72.9 (20.6)	
More than four times	31	63.2 (14.7)		78.4 (15.9)		90.3 (13.6)		55.4 (16.4)		60.1 (18.1)	
Average spending in the store / month			0.303		0.649		0.169		0.851		0.786
Less than 20€	28	68.6 (20.7)		77.1 (14.4)		87.1 (18.2)		54.0 (10.7)		69.6 (21.6)	
Between 20€ and 50€	37	60.5 (19.7)		78.9 (14.9)		85.5 (21.8)		52.4 (11.9)		67.9 (22.7)	
Between 50€ and 100€	22	59.1 (21.8)		79.5 (17.6)		77.3 (28.0)		54.0 (15.7)		71.6 (22.2)	
More than 100€	29	62.8 (16.7)		82.1 (12.4)		90.9 (19.2)		51.3 (16.0)		65.9 (14.1)	
Average spending in the store / visit			0.317		0.225		0.617		0.930		0.314
Less than 10€	21	68.6 (19.6)		76.0 (14.3)		86.3 (14.7)		53.6 (9.2)		63.1 (23.2)	
Between 10€ and 20€	46	60.9 (17.9)		78.0 (16.3)		85.6 (22.0)		53.0 (13.7)		68.2 (20.5)	
More than 20€	49	62.0 (21.3)		82.0 (12.9)		85.5 (24.5)		52.3 (14.8)		71.2 (18.8)	
Awareness of the program			<0.001		0.024		0.666		0.903		0.212
Spontaneous	22	77.3 (15.5)		82.7 (14.5)		87.5 (20.8)		52.6 (12.9)		75.0 (20.4)	
Aided	29	61.4 (18.5)		84.1 (14.3)		87.9 (20.7)		51.9 (12.1)		65.1 (20.1)	
No	65	58.5 (19.5)		76.2 (14.2)		84.0 (22.9)		53.3 (14.3)		67.9 (20.2)	
Perceived usefulness of the program			0.686		0.007		0.608		0.397		0.229
Yes	107	63.2 (19.8)		79.6 (14.3)		86.0 (21.9)		52.9 (12.9)		69.5 (19.9)	
No	4	55.0 (10.0)		60.0 (14.1)		75.0 (33.9)		45.3 (15.6)		53.1 (37.3)	
I don’t know	5	60.0 (24.5)		90.0 (10.0)		87.5 (8.8)		57.5 (22.3)		60.0 (5.6)	

Mean score (/100) of customers’ answers to a quiz assessing their knowledge in nutrition, to a quiz assessing their understanding of the labeling system, and to questions on attractiveness of promoted products. Differences in scores were analyzed based on socio-demographic characteristics, shopping profiles, and based on awareness and perceived usefulness of the program using one-way ANOVAs

#### Customers’ purchases of promoted products

As shown in Table 4, the mean contribution of promoted products to total food spending among customers in-depth survey was 13.6 %. This percentage was significantly higher among customers with food insecurity (*p* = 0.002) and customers who spend between 20 and 50 euros per month at this store (*p* = 0.044), and was significantly lower among customers who shopped less than once a month at the intervention stores (*p* = 0.036). The contribution of promoted products to overall spending on food was also positively correlated with the score measuring the placement-related attractiveness of promoted products (*p* = 0.003, *r* = 0.27; data not shown) but no significant correlations were observed between customers’ purchases of promoted products and the scores measuring the preparation-related attractiveness or taste-related attractiveness (data not shown).

**Table 4:** In-depth survey: purchases of promoted products

	Sample	Contribution of promoted products to total food spending	P
	(*n* = 116)	Mean (SD)	
Total	116	13.6 (8.2)	
Sex			0.632
Female	91	13.4 (8.1)	
Male	25	14.3 (8.7)	
Age			0.527
20–40 years	29	14.5 (10.4)	
40–60 years	51	12.6 (7.3)	
More than 60 years	36	14.3 (7.5)	
Adults at home			0.591
One	26	12.8 (11.1)	
Two or more	90	13.8 (7.2)	
Children at home			0.089
No child	45	12.3 (8.2)	
One or two	47	13.3 (9.2)	
Three or more	24	16.8 (4.9)	
Food insecurity			0.002
Yes	19	18.8 (9.8)	
No	97	12.6 (7.5)	
Occupational status			0.156
Unemployed, disabled, student	42	15.4 (9.1)	
Lower socio-professional category	30	11.2 (7.2)	
Upper socio-professional category	12	12.0 (7.6)	
Retired	32	14.1 (7.7)	
Frequency of shopping at the store			0.036
Less than once	19	9.2 (7.8)	
Two to four times	66	14.6 (7.9)	
More than four times	31	14.1 (8.5)	
Average spending in the store / month			0.044
Less than 20€	28	11.8 (9.2)	
Between 20€ and 50€	37	16.7 (10.1)	
Between 50€ and 100€	22	12.7 (4.9)	
More than 100€	29	12.0 (5.2)	
Average spending in the store / visit			0.059
Less than 10€	21	16.1 (11.8)	
Between 10€ and 20€	46	14.6 (7.9)	
More than 20€	49	11.6 (6.0)	
Awareness of the program			0.405
Spontaneous	22	15.4 (6.0)	
Aided	29	12.2 (9.6)	
No	65	13.6 (8.1)	
Perceived usefulness of the program			0.375
Yes	107	13.4 (7.9)	
No	4	19.2 (14.7)	
I don’t know	5	14.4 (9.6)	

Average contribution (%) of promoted products to the total spending on food of member-customers. Differences in purchases of promoted products were analyzed based on socio-demographic characteristics, shopping profiles, and based on awareness and perceived usefulness of the program using one-way ANOVAs

## Discussion

Our results showed that a 6-month intervention combining shelf labeling with marketing strategies to promote inexpensive food products with good nutritional quality increased awareness and understanding of the nutrition labeling system in people from disadvantaged neighborhoods and had a significant impact on purchases of some food categories.

Analysis of the purchasing data revealed that the MANGER TOP intervention did not have a significant impact on customers’ total food purchases, but did have a positive effect on purchases of fruits and vegetables and starches. This result, even though it only applies to two out of six food categories, is very encouraging in view of the difficulty in changing purchasing behavior during a 6-month in-store intervention. A review of the literature clearly shows that interventions are more likely to have an impact on sales the longer they last [32]. The positive effect on purchases observed in our study is consistent with previous research showing that interventions that include additional promotional activities are the most effective in modifying purchasing behavior [31–33].

The purchases of inexpensive foods with good nutritional quality accounted for approximately 20 % of food purchases on average. In a subset of customers for which we matched purchasing data with responses to in-depth survey, the contribution of promoted products tot total food spending was significantly higher in customers experiencing food insecurity. Indeed, our preliminary study showed, consistent with previous research in populations with a low socio-economic status [55], that price is a major determinant of food choices for residents of the 14th and 15th districts of Marseille. It is therefore not surprising that the promoted products, which we selected partly because they were inexpensive, already accounted for a non-negligible part of the purchases made by the customers included in our study. Our results therefore highlight the need to consider economic concerns in future studies of nutrition labeling in low socio-economic status populations.

According to our exit survey, 31 % of customers were aware of the labeling system, half of which exhibited spontaneous awareness. As expected, this percentage was higher among the member-customers who participated in the in-depth survey, and increased at the end of the intervention. This result emphasizes the importance of time, as well as differences in customer responsiveness when introducing a nutrition label, just as when launching a new product or service [62]. A longer intervention with an advertising plan adapted to different segments of the population may be needed to exert more substantial effects on purchases. Moreover, the communication tools were only present inside the store, and the labeling system was hung on shelves, which prevented customers from becoming accustomed to the labeling system outside of the store setting. On-pack labeling and multi-channel communication, including TV/radio/online campaigns, would probably have helped to spread and strengthen the message, as is the case for promoting brands [63, 64], and we believe that future studies should closely examine these factors.

Our in-depth survey also showed that customers’ awareness of the intervention was positively associated with their knowledge of nutrition and understanding of the labeling system, suggesting that customers who were aware of the MANGER TOP intervention were also aware of nutrition concerns in general. These results are consistent with findings from Grunert et al., who showed that customers’ knowledge of nutrition plays a major role in awareness and understanding of a nutrition labeling system [3]. Future research should consider segmenting the population to take into account differences in knowledge of nutrition and differences in responsiveness to nutrition labels, and to adapt the intervention tools and messages to these different target populations.

When looking at the scores we developed to evaluate attractiveness of promoted products, we did not identify any significant association between customers’ purchases of promoted products and their taste-related or preparation-related attractiveness scores (which were instead associated with socio-demographic characteristics such as age or gender). However, we found that customers’ purchases of promoted products were positively associated with their placement-related attractiveness score, highlighting the importance of store architecture in purchasing behavior. Indeed, in-store purchasing behavior is rarely based on an evaluative process of the customer who takes into account nutrition, price, taste, or preparation concerns. It has been widely demonstrated that the store environment and the time available for shopping have a strong impact on shopping behaviors, which can lead to both failure to make the intended purchases and impulse buying [65–67]. It is noteworthy that this seems to be also the case for inexpensive foods with good nutritional quality. To promote healthy foods in a supermarket setting, future interventions should not only take into account nutrition, price, taste, and preparation concerns, but also explore nudge theory in store setting to influence shoppers’ food choices [68].

## Strengths and limitations

This study is not without limitations. First, only four grocery stores were included in the study. In fact, the social marketing approach required regular attendance of the research team in the stores to implement the intervention by installing, following up and adapting the intervention materials, ie shelf labels, posters, leaflets, prime placement, taste-testing booth. To maintain the desired level of quality for this intervention, we could not extend it to more than two intervention stores and two control stores. Second, we could not compare the stores based on quantitative data. Indeed, we did not have access to any quantitative data from the stores (size, number of employees, whole store sales, number of visits per day) as the retail chain managers considered them as critical. We thus relied on the retail chain managers who selected the stores for us and assigned them to intervention/control arms. Third, we could not know whether the intervention store customers and control store customers were comparable or not. Since in-depth surveys were not administered at the control stores, we had demographic data, shopping profiles, and knowledge of nutrition from intervention stores customers only. We thought we could collect demographics from both intervention stores and control stores customers with the loyalty cards, but it appeared that customers rarely fill the registration form when they subscribe to the loyalty program. Demographic data, shopping profiles, and knowledge of nutrition from the customers would have be helpful to compare populations from the two groups, especially since we observed baseline differences in purchases of inexpensive foods with good nutritional quality between intervention and control stores. Fourth, we selected only member-customers for purchasing data analysis and in-depth surveys. Using data from loyalty card was the only way for us to get purchasing data at the individual level and to track purchases over time. Therefore, this resulted in sample sizes (6,625 customers for the purchasing data analysis and 144 customers for the in-depth survey) and findings may not be applicable to non member-customers.

Despite these limitations, strengths should also be noted. First, we have assessed the impact of the MANGER TOP on purchases at the customer level rather than on sales at the store level. This seemed to us more relevant since the objective of the intervention was to improve customers’ purchasing behaviors rather than increasing stores sales of promoted products. Second, using data from the loyalty cards allowed us to track customers’ purchases from one year to another. We could thus compare customers’ purchases based not only on the type of store (intervention/control) but also the year (2013/2014). In contrast to a time-series estimate or a cross-section estimate, this statistical technique called « difference in differences » is intended to mitigate the effects of extraneous factors, and mimics an experimental research design using observational study data. Third, we were able for a subset of customers to match the purchasing data with their responses to questions posed as part of the in-depth survey. This allowed us to link purchasing behaviors with other individual variables such as demographic data, shopping profiles, and knowledge of nutrition. Fourth, we sought to measure the impact of MANGER TOP on customers through other indicators than only purchasing data, including awareness and perceived usefulness of the program, as well as understanding of the labeling system and attractiveness of promoted products. We also distinguished spontaneous awareness from aided awareness to draw attention to subconscious responsiveness, as it is well documented in the marketing literature [69]

## Conclusion

Our study demonstrated the effectiveness of a shelf-labeling intervention aimed at increasing the visibility and attractiveness of inexpensive foods with good nutritional quality in disadvantaged neighborhoods. The MANGER TOP intervention not only increased customers’ purchases of promoted products from two food categories, namely fruits and vegetables and starches, but also raised awareness of the labeling system over time and reached some customers by improving their understanding of the labeling system. These positive results may be due to the marketing strategy principles that were included in the development of the intervention, and could be further improved with a longer intervention time and on-pack labeling to exert significant effects on purchases.
